# Screening of mosquitoes for filarioid helminths in urban areas in south western Poland—common patterns in European *Setaria tundra* xenomonitoring studies

**DOI:** 10.1007/s00436-018-6134-x

**Published:** 2018-12-08

**Authors:** Katarzyna Rydzanicz, Elzbieta Golab, Wioletta Rozej-Bielicka, Aleksander Masny

**Affiliations:** 1 Department of Microbial Ecology and Environmental Protection, Institute of Genetics and Microbiology, University of Wrocław, Przybyszewskiego 63/77, 51-148 Wrocław, Poland; 2 Department of Parasitology, National Institute of Public Health – National Institute of Hygiene, Chocimska 24, 00-791 Warszawa, Poland; 3 Department of Influenza Research, National Influenza Center, National Institute of Public Health – National Institute of Hygiene, Chocimska 24, 00-791 Warszawa, Poland

**Keywords:** Culicidae, *Aedes*, *Culex*, *Setaria tundra*, *Dirofilaria* spp., Molecular xenomonitoring, Xenomonitoring, Filarioid helminths, Wrocław, Poland, Europe, PCR, Real-time PCR, Mosquito, Cytochrome C oxidase I, COI, Cytochrome oxidase, Phenology, Vector, Parasite

## Abstract

In recent years, numerous studies screening mosquitoes for filarioid helminths (xenomonitoring) have been performed in Europe. The entomological monitoring of filarial nematode infections in mosquitoes by molecular xenomonitoring might serve as the measure of the rate at which humans and animals expose mosquitoes to microfilariae and the rate at which animals and humans are exposed to the bites of the infected mosquitoes. We hypothesized that combining the data obtained from molecular xenomonitoring and phenological studies of mosquitoes in the urban environment would provide insights into the transmission risk of filarial diseases. In our search for *Dirofilaria* spp.-infected mosquitoes, we have found *Setaria tundra*-infected ones instead, as in many other European studies. We have observed that cross-reactivity in PCR assays for *Dirofilaria repens*, *Dirofilaria immitis*, and *S. tundra* COI gene detection was the rule rather than the exception. *S. tundra* infections were mainly found in *Aedes* mosquitoes. The differences in the diurnal rhythm of *Aedes* and *Culex* mosquitoes did not seem a likely explanation for the lack of *S. tundra* infections in *Culex* mosquitoes. The similarity of *S. tundra* COI gene sequences found in *Aedes vexans* and *Aedes caspius* mosquitoes and in roe deer in many European studies, supported by data on *Ae. vexans* biology, suggested host preference as the most likely cause of the mosquito genus-biased infections. High diversity of the COI gene sequences isolated in the city of Wroclaw in south western Poland and the presence of identical or almost identical sequences in mosquitoes and roe deer across Europe suggests that *S. tundra* has been established in most of Europe for a very long time.

## Introduction

Filarial nematodes are parasites of tissues and body cavities of all classes of vertebrates other than fishes (Anderson 2000) and pose a threat to humans, domestic animals, and wildlife (WHO 2007). The super family Filarioidea consists of the families Filariidae, Setariidae as well as Onchocercidae, and all filariae are transmitted by hematophagous arthropods (Anderson 2000).

The xenomonitoring of mosquitoes for filarioid helminths has been gaining popularity in the studies of *Dirofilaria* spp. (Czajka et al. 2012; Latrofa et al. 2012; Bocková et al. 2013; Czajka et al. 2014; Kronefeld et al. 2014; Silbermayr et al. 2014; Rudolf et al. 2014; Zittra et al. 2015; Kemenesi et al. 2015; Șuleșco et al. 2016a, b; Kurucz et al. 2016; Masny et al. 2016; Ionică et al. 2017). The range of conclusions drawn from molecular xenomonitoring depends on the ability to adequately sample the vector population, accurately determine the infection status in the vector, and link infections in the vector population to infections in the human or animal populations. It was indicated previously that in order to accurately calculate the risk of disease transmission to vertebrates, it is necessary to identify the vector species in a given area and estimate the abundance and the distribution of the potential vector species (Norris 2002). The entomological monitoring of filarial infections in mosquitoes and molecular xenomonitoring in particular may serve as the measures of the rate at which animals expose mosquitoes to microfilariae and the rate at which animals and humans might be exposed to the bites of the infected mosquitoes (Chambers et al. 2009). Detection of *Setaria tundra* and *Dirofilaria* spp. by PCR xenomonitoring of mosquitoes may serve as an example of molecular xenomonitoring applicability for indirect filariae presence detection in local populations of the vertebrate hosts (Czajka et al. 2012; Latrofa et al. 2012; Bocková et al. 2013; Czajka et al. 2014; Kronefeld et al. 2014; Silbermayr et al. 2014; Rudolf et al. 2014; Zittra et al. 2015; Kemenesi et al. 2015; Șuleșco et al. 2016a, b; Kurucz et al. 2016; Masny et al. 2016). Both *S. tundra* and *Dirofilaria repens* were detected in the same xenomonitoring studies (Czajka et al. 2012; Kronefeld et al. 2014; Zittra et al. 2015; Kemenesi et al. 2015), and in most cases, the detection of *S. tundra* was a result of xenomonitoring mosquitoes for *Dirofilaria* spp. Animal and human dirofilariosis, caused by *D. repens*, has become a filarial disease established in many European countries (Simón et al. 2012; Sałamatin et al. 2013; Harizanov et al. 2014; Antolová et al. 2015; Fuehrer et al. 2016), and the veterinary and medical importance of the parasite was well studied. The infections caused by *S. tundra* have not been investigated in such detail as the *Dirofilaria* spp. infections. The parasite *S. tundra* was documented in many European countries: in reindeer in the former USSR (Rajewsky 1928), in reindeer from Sweden (Rehbinder 1990) and Norway (Kummeneje 1980), in roe deer from Bulgaria (Yanchev 1973) and Germany (Büttner 1978; Rehbein et al. 2000; Czajka et al. 2012), and roe deer from Italy (Favia et al. 2003), Poland (Bednarski et al. 2010; Kowal et al. 2013; Kuligowska et al. 2015), and Denmark (Enemark et al. 2017). The veterinary importance of *S*. *tundra* was described, an outbreak of peritonitis with significant economic losses in semi-domestic reindeer (*Rangifer tarandus tarandus*) in Finland in 1973 and in 2003–2005 and in moose (European elk, *Alces alces*) in Lapland in 1989 (Laaksonen et al. 2008, 2009). To date, there is only scant information on the transmission and specific vectors of *S. tundra*. The authors of previous studies described the following mosquitoes as vectors of *Setaria* sp.: *Aedes aegypti* Linnaeus (Wajihullah 1981, 2001), *Aedes canadensis* Theobald (LeBrun and Dziem 1984), *Aedes caspius* Pallas (Pietrobelli et al. 1998), *Aedes communis* De Geer, *Aedes excrucians* Walker (Laaksonen et al. 2009), *Aedes punctor* Kirby, *Aedes togoi* Theobald (Hagiwara et al. 1992), *Aedes vexans* Meigen, *Aedes sierrensis* Ludlow (Prestwood and Pursglove 1977), *Aedes sticticus* Meigen (Czajka et al. 2012), *Anopheles claviger* Meigen, *Anopheles hyrcanus* Pallas, and *Anopheles sinensis* Wiedemann (Laaksonen 2010). For *D. repens*, competent mosquito vector species were confirmed, by experimental infection leading to the development of the third stage infective larvae: *Anopheles atroparvus* Van Thiel, *Culex pipiens* biotype *molestus* Forskal, and *Ae. aegypti* (Kuzmin et al. 2005). Other authors described *Ae. vexans* as a potential vector of *D. repens* (Bocková et al. 2015; Rudolf et al. 2014).

The filarial transmission, including *S. tundra* and *D. repens*, is highly dependent on the life span of the female mosquito vectors, with availability of the breeding sites, survival of the adult mosquitoes depending on both temperature and humidity as well as on the host feeding pattern of mosquito vectors (Clements 1963; Börstler et al. 2016). Warm summers improve transmission and genesis of disease outbreaks by favoring the development of filarioid helminths in their mosquito vectors (Genchi et al. 2009; Laaksonen et al. 2009). Diurnal activity of the mosquito species of medical and veterinary importance was monitored in many regions of Europe such as Croatia, the Czech Republic, France, and Sweden (Merdič and Boca 2004; Šebesta et al. 2011; Ponçon et al. 2007; Jaenson 1988). Precise knowledge of the relative abundance and phenology of the relevant mosquito species is essential for establishing the biology and the ecology of mosquito-borne filarial parasites.

The aims of our study were to identify the mosquito species in which filarial infections occur and to investigate the diurnal activity patterns of the mosquito species infected with filarioid helminths for assessing the routes and time of filarial nematode infection and potential transmission in urban ecosystems.

## Materials and methods

### Study area and adult mosquito surveillance

The collection of adult mosquitoes was carried out in the irrigation fields located in the northeastern part of Wrocław (SW, Poland) area in August and September 2012. This area was constructed in 1890 in the Odra River Valley to provide wastewater treatment before disposal into the river system (Rydzanicz et al. 2011). The system consists of sewage reservoirs, sewage canals, ancient river meanders, fields intermittently providing standing water bodies for varying periods of time, and underground drainage pipe systems. Floodwater mosquitoes, mainly *Ae. caspius* and *Ae. vexans*, emerge from these fields in huge numbers every summer when there is intermittent flooding with wastewater entering infiltration fields while sewage canals support development of *C. pipiens* s.l. (Linnaeus)/*Culex torrentium* Martini (Weitzel et al. 2015)*.*

Five CDC/EVS traps with carbon dioxide (BioQuip, Products INC, Rancho Dominiquez, CA, USA) supplemented by dry ice as an attractant for host-seeking female mosquitoes were placed in different locations (Osobowicki forest 51° 09′ 11′′ N, 16° 59′ 39′′ E; shelter for homeless animals 51° 09′ 08′′ N, 16° 59′ 56′′ E; sewage polders 51° 09′ 20′′ N, 17° 00′ 08′′ E and 51° 09′ 31′′ N, 16° 59′ 48.27′′ E; human settlement 51° 09′ 07′′ N, 16° 59′ 39′′ E). In the morning, mosquitoes collected at the study sites were transported to the laboratory for morphological identification on chill tables according to standard taxonomic keys to the mosquitoes of Europe (Becker et al. 2010) and stored at − 20 °C until DNA extraction. The classification of tribe Aedini was followed after Wilkerson et al. (2015).

Data for the diurnal rhythm of mosquitoes were collected in the same locations on the irrigation fields from August 7 to 8, 2012. Five CDC-CO_2_ traps were deployed at 1 p.m. Central European Summer Time and mosquitoes were collected for 24 h and the numbers of females caught in each hour period were summed. Temperature and humidity were recorded every hour using a portable thermo-hygrometer (LB-702, Lab-EL, Poland).

### DNA analysis

Randomly selected mosquitoes (only limited number of all collected mosquitoes was subjected to molecular analyzes) were divided into pools of 10 insects and were subjected to DNA extraction, 340 *Ae. vexans*, 610 *Ae. caspius*, and 1000 *Cx. pipiens* s.l*.*/*Cx. torrentium* mosquitoes. The DNA was extracted using the guanidinium thiocyanate protocol (Boom et al. 1999) modified for DNA extraction from mosquitoes (Masny et al. 2016). The DNA was subjected to PCR for amplification of the cytochrome oxidase subunit one gene (COI) fragments. Four types of PCR assays were used: two PCR assays for *Dirofilaria* spp. detection and two PCR assays for *S. tundra* detection. For *Dirofilaria* spp. detection screening, PCR assay (for the detection of many filarial nematodes species) with primer pair RepIm-F/RepIm-R2 (Masny et al. 2016) and confirmatory PCR with RepIm-F0/RepIm-R0 primers for *Dirofilaria* spp. detection (Masny et al. 2016) were performed. For *S. tundra* detection, two distinct PCR assays with primer pairs: Set-F0/RepIm-R0 and Set-Fs/Set-R2 were performed. The primer pairs Set-F0 (5’-TCAGGCTAGTATGTTTGTTTGAACTTCTTATTT-3′) and RepIm-R0 (Masny et al. 2016), Set-Fs (5’-AGTAGTTGAACTTTTTATCCTCCTCT-3′), and Set-R2 (5’-GACCAAATAAACGATCCTTATCAG-3′) perfectly matched numerous *S. tundra* sequences from the GenBank. The primers Set-Fs, Set-R2, and Set-F0 were a redesigned version of universal primers: RepIm-Fs, RepIm-R2, and RepIm-F0 used for *Dirofilaria* spp. screening (Masny et al. 2016); the *Setaria* DNA mismatching bases had been indicated in the multiple sequence alignment of *S. tundra*, *D. repens*, *Dirofilaria immitis*, and *Acanthocheilonema reconditum*, and in the species-specific mismatches presented in tables in previous studies (Masny et al. 2016). The primer residues mismatching the *S. tundra* sequence were substituted with bases perfectly matching the *S. tundra* COI gene sequence; thus, RepIm-Fs primer was converted to Set-Fs primer and RepIm-R2 primer was converted to Set-R2 primer.

To every PCR reaction, 1 μl of DNA solution was added and the final volume of all PCR samples was 20 μl, 1× concentrated SsoFastTM EvaGreen Supermix, 500 nM each of the primers. The thermal profile of PCR with primer pair Set-F0/RepIm-R0 started with initial denaturation at 95 °C 3 min followed by 45 cycles of the two-step reaction, 95 °C 15 s, 60 °C for 40 s. Signal was acquired in the green channel at the end of incubation at 60 °C in each cycle. The thermal profile of PCR with primer pair Set-Fs/Set-R2 was as follows: initial denaturation 95 °C 3 min followed by 45 cycles of the three-step reaction, 95 °C 15 s, 64 °C 20 s, and 72 °C 20 s. Signal was acquired in the green channel at the end of incubation at 72 °C at each cycle. The positive controls were samples of *S. tundra*, *D. repens*, and *D. immitis* DNA. The negative control was DNA from mosquito larvae.

The PCR products obtained on the template of DNA extracted from mosquitoes were subjected to Sanger sequencing. The chromatograms were assembled using the CLC Main Workbench 7.7.1 software. Assembled sequences were subjected to NCBI nucleotide blast search. The analyses of PCR primer similarity to filariae DNA sequences deposited in the GenBank were performed with NCBI BLAST.

## Results

In total, 7392 mosquito females were collected from August to September 2012 in five locations at the irrigation fields in Wrocław. During hourly observations of the mosquito activity, we collected 5442 mosquito females among which three species were dominant *Ae*. *caspius* (*n* = 3657), *Ae*. *vexans* (*n* = 1360), and *Cx*. *pipiens* s.l./Cx. *torrentium* (*n* = 358). Other sympatrically occurring species recorded by the traps were *An. claviger* s.l. Meigen, 1804 (*n* = 6), *Anopheles maculipennis* s.l. Meigen, 1818 (*n* = 6), *Aedes cantans* Meigen, 1818 (*n* = 9), *Aedes cinereus* Meigen, 1818 (*n* = 18)*, Ae. sticticus* Meigen, 1838 (*n* = 18) *Culiseta annulata* Schrank, 1776 (*n* = 4), and *Coquillettidia richiardii* Ficalbi, 1889 (*n* = 6)*.*

**Fig. 1:**
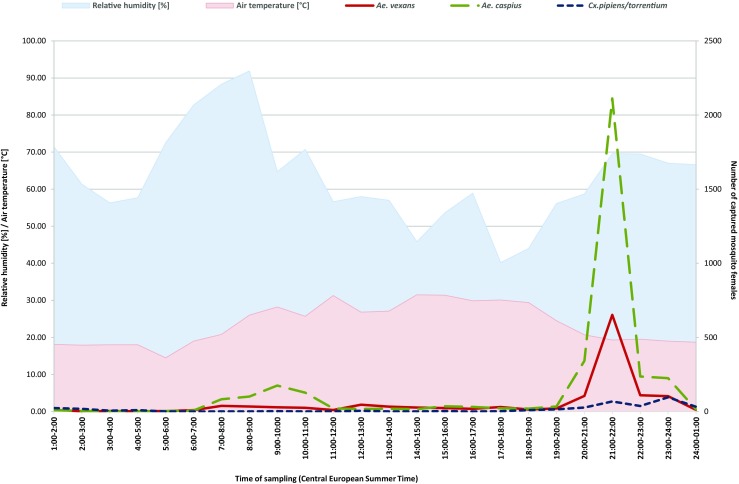
Relationship between variability of daily air temperature, relative humidity, and activity of dominant mosquito species in irrigation fields, Wrocław (total per sampling trip summed from the five locations) from 7th to 8th of August, 2012

Mosquito females of the dominant species exhibited activity throughout the 24 h; however, the first peak of *Ae. caspius* and *Ae. vexans* was observed between 9 a.m. and 10 a.m. (206 specimens) when the air temperature and the relative humidity reached 28.2 °C and 64.7%, respectively (Fig. 1). The number of mosquito females increased significantly between 8 p.m. and 11 p.m. (2832 specimens) when the air temperature was decreasing to the minimum of 19.3 °C and relative humidity reached 69.6% (Fig. 1). Additionally, *Ae. caspius* showed increasing activity from 7 a.m.to 10 a.m. (507 specimens) when the air temperature varied from 20.8 to 31.1 °C and relative humidity was decreasing from 88.3 to 56.6%. (Fig. 1). Female *Cx*. *pipiens* s.l. cannot be distinguished from *Cx*. *torrentium*, and both were counted together with a total amount of 358 specimens captured. These species showed increasing nocturnal activity between 7 p.m. and 1 a.m. (Fig. 1).

The DNA was extracted from 1950 mosquitoes collected on two separate days, August 7, 2012 and September 4, 2012. All mosquitoes were divided into pools of 10 specimens collected on the same date, belonging to the same species except *Cx. pipiens* s.l. divided into 80 pools of 10 mosquitoes and 4 pools of 50 mosquitoes. The following are numbers of mosquitoes collected on August 7, 2012 and September 4, 2012, 420 and 190 *Ae. caspius*, 130 and 210 *Ae. vexans*, and 860 and 140 *Cx. pipiens* and/or *Cx. torrentium* mosquitoes were subjected to DNA extraction, respectively. One pool of *Ae. vexans* and one pool of *Ae. caspius* collected on September 4, 2012 were positive for *S. tundra*. No infections with *D. repens* or *D. immitis* were detected using the previously described approach employing *Dirofilaria* spp. screening PCR with primer RepIm-F and RepImR2 and confirmatory PCR with primer pairs RepIm-F0 and RepIm-R0 (Masny et al. 2016). The results of sequencing confirmed that *S. tundra* DNA was present in three mosquito pools collected on September 4, 2012 (Table 1). The PCR, with the primer pair RepIm-F0 and RepIm-R0, for *D. repens* detection allowed detection of two mosquito pools containing *S. tundra* DNA (Table 2). Using two primer pairs RepIm-R0/Set-F0 and Set-Fs/Set-R2 designed to perfectly match the *S. tundra* COI gene, a total of three mosquito pools were found to be positive (Table 2). Those three positives included two positive pools initially detected by PCR assays for *D. repens* detection and one pool detected by *S. tundra*-specific primer pairs, only (Table 2). The positive pools contained 10 mosquitoes each: *Ae. caspius* (one pool) and *Ae. vexans* (two pools). At least five variants of COI gene sequences were detected. The sequences had a high level of similarity or were identical to *S. tundra* sequences obtained from roe deer (*Capreolus capreolus*) and *Ae. vexans* or in one case from mosquitoes of unidentified species (Table 1). The primer pairs Set-F0/RepIm-R0 and Set-Fs/Set-R2 had perfect binding sites within the *S. tundra* COI gene sequences described in Table 1: KX599456, KX599455, KU508985, KU508984, KU508983, KM452922, KF692103, and AM749298. The primer pairs Set-F0/RepIm-R0 and Set-Fs/Set-R2 had perfect binding sites within the GenBank sequence KP760209 obtained from *S. tundra* isolated from *R. tarandus* from Finland (Lefoulon et al. 2015). The single sequence obtained from *S. tundra* isolated from *C. capreolus* from Denmark [KU508982] was found to contain perfect binding sites for the Set-Fs/Set-R2 primer pair and have a single mismatch with RepIm-R0 primer from the Set-F0/RepIm-R0 primer pair within the primer binding site. Two identical sequences of *S. tundra* COI gene fragments obtained from mosquito pools collected in central Poland [KM370867] (collected on July 8, 2012) and [KM370868] (collected on July 3, 2012) perfectly matched KM452922, KF692103, and AM749298 sequences identified in this study (Table 1). The single *S. tundra* sequence from a worm isolated from *C. capreolus* in Finland was found in GenBank [EF661849]. The latter sequence overlapped only with a short region of the sequences identified in this study and the sequences of *S. tundra* COI gene [KP760209, KX599455, KX599456] which were described in various European studies and included in the phylogenetic analysis of European *S. tundra* COI gene sequences performed by Angelone-Alaasad et al. 2016.

**Table 1:** Similarity of the detected COI gene sequences of *S. tundra* to the sequences deposited in GenBank

Study specimens (pools of ten mosquitoes)	Similarity (%)	Host	Specimen characteristics	Collection site	Reference
KY246313 (7a2) *Aedes caspius*	KU508983 (99%)	*Capreolus capreolus*	Liver cyst	Denmark	Enemark et al. 2017
	KM452922 (99%)^*4^	*Aedes vexans*	Mosquito^*1^	Hungary, Szeged	Zittra et al. 2015
	KF692104 (99%)^*4^	*Aedes vexans*	Mosquito pool	Germany, Radolfzell	Kronefeld et al. 2014
	KF692103 (99%)	*Aedes vexans*	Mosquito pool	Germany, Braunschweig	Kronefeld et al. 2014
	AM749298 (99%)^*4^	*Capreolus capreolus*	Worm specimen^*2^	France	Ferri et al. 2009
	AJ544874 (99%)	not specified	Not specified	not specified	Casiraghi et al. 2004
KY246312 (7a1) *Aedes caspius*	KF692105 (99%)	*Aedes vexans*	Pool	Germany, Regensburg	Kronefeld et al. 2014
KY246309 (W10) *Aedes vexans*	KU508983 (100%)	*Capreolus capreolus*	Liver cyst	Denmark	unpublished
	KF692104 (100%)	*Aedes vexans*	Mosquito pool	Germany, Radolfzell	Kronefeld et al. 2014
KY246310 (W7-1) *Aedes vexans*	KX599456 (100%)	*Capreolus capreolus*	Peritoneal cavity	Spain, La Alcarria	Angelone-Alasaad et al. 2016
	KX599455 (100%)	*Capreolus capreolus*	Peritoneal cavity	Spain, La Alcarria	Angelone-Alasaad et al. 2016
	KU508985 (100%)	*Capreolus capreolus*	Peritoneal cavity	Denmark	Enemark et al. 2017
	KU508984 (100%)	*Capreolus capreolus*	Peritoneal cavity	Denmark	Enemark et al. 2017
KY246311 (W7-2) *Aedes vexans*	KM370867 (100%)	Mosquito	Mosquito pool^*3^	Poland, Kanie	Masny et al. 2016
	KM452922 (100%)^*4^	*Aedes vexans*	Mosquito^*1^	Hungary, Szeged	Zittra et al. 2015
	KF692103 (100%)^*4^	*Aedes vexans*	Mosquito pool	Germany, Braunschweig	Kronefeld et al. 2014
	AM749298 (100%)^*4^	*Capreolus capreolus*	Worm specimen^*2^	France	Ferri et al. 2009

Three pools of ten mosquitoes: 7a, 10, and 7 contained *S. tundra* DNA. Study specimens: GenBank accession number, isolate identifier in brackets followed by sequence code number, infected mosquito species. Similarity: the level of sequence similarity to the sequences from GenBank revealed by NCBI BLAST

^*1^HU91 isolate, single blood fed mosquito

^*2^Bain, O. MI_FR_ST_SET1 (MNHN, Museum National d’histoire Naturelle, Paris, France

^*3^Isolate 08072012-48 pool of ten mosquitoes of undetermined species, Poland

^*4^*S. tundra* sequences (KM370867; KM370868) from central Poland perfectly match those sequences

**Table 2:** PCR primers

PCR	Primers		Positive pools
Screening *Dirofilaria*	RepIm-F RepIm-R2	GGTAATCCTTT**g**TTGTATCAGCA	3/3
		GACCAAA**c**AAACGATCCTTATCAG	
Confirmatory *Dirofilaria*	RepIm-F0 RepIm-R0	TCAG**at**TAGTATGTTTGTTTGAACTTCTTATTT	2/3
		ACAGCAATCCAAATAGAAGCAAAAGT	
Confirmatory 1 *Setaria*	Set-F0 RepIm-R0	TCAG**GC**TAGTATGTTTGTTTGAACTTCTTATTT	3/3
		ACAGCAATCCAAATAGAAGCAAAAGT	
Confirmatory 2 *Setaria*	Set-Fs Set-R2	AGTAGTTGAACTTTTTATCCTCCT**C**T	3/3
		GACCAAA**T**AAACGATCCTTATCAG	

Bold lower case are the bases mismatching the *S. tundra* COI gene. Gray, Bold uppercase are bases mismatching *D. repens* DNA

*Screening Dirofilaria* screening PCR designed for *Dirofilaria* spp. detection, *Confirmatory Dirofilaria* confirmatory PCR for *Dirofilaria* spp*.* detection, *Confirmatory Setaria* confirmatory PCRs for *S. tundra* detection

## Discussion

### Combining molecular and vector biology data

Our initial goal was to detect *D. repens*; therefore, one of the mosquito traps was located close to an animal shelter for dogs. Infections with *D. repens* were previously confirmed in mosquitoes in central Poland (Masny et al. 2016). There was no information on the *Dirofilaria* spp. infection status of the dogs in the vicinity of our xenomonitoring study site; however, in the Lower Silesia region were our site was located, approximately 3% of dogs were infected with *D. repens* (Demiaszkiewicz et al. 2014). We expected *S. tundra* presence, based on the results of a study from central Poland (Masny et al. 2016). However, in the latter study, the species of filarioid helminth-infected mosquitoes was not determined. In Hungary (Zittra et al. 2015*)* and Germany (Kronefeld et al. 2014), *D. immitis* was detected in *Culex* spp.*;* in Serbia, both *D. repens* and *D. immitis* were detected in *Cx. pipens* (Kurucz et al. 2016); and in Belarus, *D. repens* was detected in *Culex* mosquitoes (Șuleșco et al. 2016a), which proved that those mosquitoes fed on *Dirofilaria* spp. hosts in the natural environment in central and eastern Europe. Infections with *Dirofilaria* spp. were detected in *Ae. vexans* and/or *Ae. caspius* mosquitoes in Hungary (Kemenesi et al. 2015; Zittra et al. 2015), Germany (Kronefeld et al. 2014), Serbia (Kurucz et al. 2016), Slovakia (Bocková et al. 2013), the Czech Republic (Rudolf et al. 2014), and Italy (Latrofa et al. 2012).

We decided to investigate a sample of all collected mosquitoes consisting of approximately 50% of *Aedes* sp. and *Culex* sp. mosquitoes, to increase the chances of *Dirofilaria* spp. detection in the competent vector (*Culex* sp.) or postulated vector (*Aedes* sp.). We did not find any infections with *Dirofilaria* spp. in the investigated mosquitoes which might result from lack of *Dirofilaria* spp.*-*infected vertebrates in the investigated area, low infection rate of the definitive hosts with *D. repens* which would correspond to low infection rate of mosquitoes, or from the absence of infected mosquitoes on the collection dates. In central Poland where the infection rate of dogs was approximately 20–25% (Osińska et al. 2014; Demiaszkiewicz et al. 2014), the infection rate of mosquitoes reached only approximately 3%, with big variation between collection days (Masny et al. 2016. Only 3% of dogs in the Lower Silesia region were infected with *D. repens* (Demiaszkiewicz et al. 2014); therefore, it is probable that the infection rate of mosquitoes with *D. repens* was so low that no infected mosquitoes were present in the investigated samples. Previous findings indicate that the PCR xenomonitoring applicability to filarial nematodes detection in mosquitoes might be limited in the regions with low prevalence of dirofilariosis in dogs (Masny et al. 2016). In the current study, we detected only *S. tundra* in the investigated mosquitoes. Furthermore, *S. tundra* was detected only in *Ae. vexans* and *Ae. caspius*. In the course of reviewing the previously published data, we realized that in other European studies, *S. tundra* parasites were found in the following mosquitoes: *Ae. vexans* (Kronefeld et al. 2014; Zittra et al. 2015) and *Ae. annulipes* Meigen, *Ae. rossicus* Dolbeshkin, Goritshkaya and Mitrofanova and *C. richiardii* Ficalbi (Kemenesi et al. 2015). Both *Ae. vexans* and *Ae. caspius* are polycyclic and breed in inundated areas of rivers, lakes, or various constructed wetlands such as irrigation or rice fields (Becker et al. 2010; Rydzanicz et al. 2011). Due to the “hatching in installments” strategy, *Ae*. *caspius* and *Ae*. *vexans* can become very abundant. Their ability to bite humans and other mammals in rural and urban areas as well as to migrate for long distances give these mosquito species additional attributes of ideal vectors of various pathogens. In the search for the probable cause of lack of *S. tundra* infections in *Culex* mosquitoes, the daily activities of *Aedes* and *Culex* mosquitoes were compared.

In our study, the diurnal rhythm of *Aedes* and *Culex* mosquitoes overlapped between 07 p.m. and 01 a.m. The difference were the peaks of activity; the peak common to both *Aedes* and *Culex* species was around 08 p.m. and 10 p.m.; *Culex* mosquitoes had second peak of activity between 11 p.m. and 00 a.m. However, the time of nocturnal *Aedes* and *Culex* mosquito species activity overlapped in general (Fig. 1); only the peaks of the activity overlapped incompletely. Another difference in the diurnal rhythm of *Culex* and *Aedes* species was a slight increase of activity of *Ae. caspius* and to a lower extent *Ae. vexans*, between 07 and 12 a.m. (Fig. 1). The latter may be explained by high *Ae. caspius* resistance to heat and drought. According to Petrić (1989), *Ae*. *caspius* females actively search for blood at temperatures ranging from 11.5 to 36.0 °C and relative humidity ranging from 47 to 92%, and this behavior of *Ae*. *caspius* was also observed in Wrocław. The observed high abundance and similar activity of *Ae. caspius* and *Ae. vexans* throughout the day corresponded to the greatest animal and human (farmers, forestry workers, gardeners, inhabitants) activity periods. However many *Aedes* species are also active at day time but only in the shadow (Becker et al. 2010). In our study, sampling mosquitoes for diurnal rhythm analysis was performed on a single day; however, similar results of high incidence and daily rhythm of *Ae*. *vexans* have been presented by Šebesta et al. (2011) in southern Moravia. To summarize, there were slight differences in the diurnal activity of *Aedes* and *Culex* species; however, those were too small to be a convincing explanation of the lack of *S. tundra* infections in *Culex* mosquitoes.

**Fig. 2:**
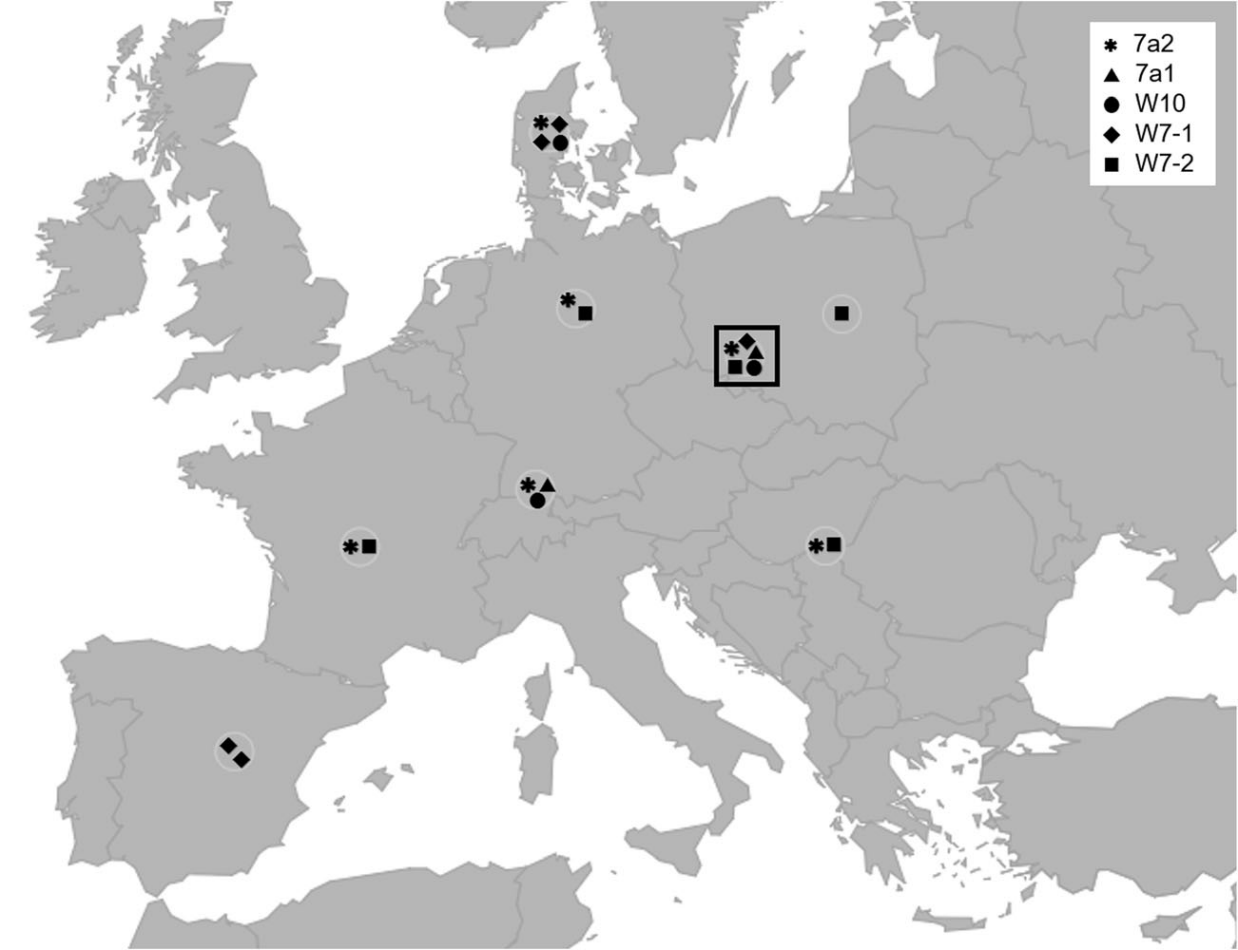
Geographic distribution of *S. tundra* COI gene variants. Five of the sequences of the *S. tundra* COI gene obtained from three pools of ten *Aedes* mosquitoes were deposited in GenBank. *Aedes caspius* pool 7a (KY246312 code 7a1, KY246313 – 7a2) and *Aedes vexans* pools W7 and W10 (KY246309 – W10, KY246310 code – W7-1, KY246311 – W7-2). On the map, the collection sites of the biological material from which the sequences with 99% identities with 7a or 7a1 sequences and 100% identities with W10, W7-1, and W7-2 sequences were obtained are indicated by circles. Collection site of the material from this study is indicated by a rectangle

The results of *S. tundra* COI gene sequence comparisons to those obtained in other mosquito xenomonitoring studies and in *S. tundra* specimen studies indicated a possible explanation of the lack of *S. tundra* infections in *Culex* mosquitoes. *Setaria tundra* COI gene sequences we detected in the mosquitoes were identical or had at least 99% similarity to the sequences of *S. tundra* obtained from two sources: *Ae. vexans* mosquitoes and roe deer (*C. capreolus*) from various regions of Europe (Table 1 and Fig. 2). In Poland, Germany, Denmark, Bulgaria, and Spain, *S. tundra* was detected in roe deer. This fact suggested that the probable definitive host of *S. tundra* detected in mosquitoes might have been *C. capreolus*. In a German study, host preference of mosquitoes including *Ae. vexans* and *Cx pipens* s.l. (Börstler et al. 2016) was discussed. According to the authors, both *Ae. vexans* and *Cx. pipens* form *pipiens* and *Cx. torrentium* feed on mammals. However, according to the data presented in the study, *Cx. pipens* form *pipiens* rarely fed on *C. capreolus* (in 3 of 22 trapping sites) in contrast to *Ae. vexans* which mostly fed on *C. capreolus* (in 19 of 23 trapping sites). Frequent feeding of *Ae. vexans* on *C. capreolus* revealed in the study performed in Germany (Börstler et al. 2016) and identical *S. tundra* COI gene fragment sequences (Table 1) found in *C. capreolus* and *Ae. vexans* from Poland (in this study and in the study by Masny et al. 2016), Germany (Kronefeld et al. 2014), Denmark (Enemark et al. 2017), Hungary (Zittra et al. 2015), France (Ferri et al. 2009), Italy (Favia et al. 2003), and Spain (Angelone-Alasaad et al. 2016) are an indication that *Ae. vexans* acquires *S. tundra* by feeding on *C. capreolus*. Lack of *S. tundra* infections in *Culex* mosquitoes could be explained by their rare feeding on *C. capreolus* (Börstler et al. 2016). Therefore, the host preference might be the most likely explanation why *S. tundra* has not been found in *Culex* mosquitoes in most European xenomonitoring studies (Czajka et al. 2012; Kronefeld et al. 2014; Zittra et al. 2015) with the exception of the very recent Austrian study (Übleis et al. 2018). The results of the phenology, feeding patterns, and molecular xenomonitoring studies constitute complementary sources of information on parasite biology, and the results of independent European studies seem to be mutually confirmatory with respect to *Ae. vexans* vector and host preference. Another factor that might influence the rate of filariae transmission is daily fluctuations of filarioid nematodes in the peripheral blood of the infected hosts. According to the laboratory experiments on the periodicity of *D. immitis* microfilariae, the number of microfilariae in peripheral blood of dogs increases from 4 p.m. in the afternoon and reaches the highest number at 4 a.m. (Miterpáková et al. 2016). The increase in *Dirofilaria* spp. microfilariae numbers overlaps with the increased activity of *Ae. vexans* and *Ae caspius* recorded in our study; the number of mosquito females increased significantly between 8 and 11 p.m. and *Ae. vexans* was confirmed to be infected with *Dirofilaria* spp. in several regions of Europe. We were unable to find any data on the fluctuations of *S. tundra* microfilaria numbers in peripheral blood of animals. Such information would be useful in the studies of the biology and the epidemiology of *S. tundra* similar to those performed for *D. immitis* (Miterpáková et al. 2016). The information on the activity patterns of free ranging animals is scant; however, the activity patterns of roe deer have been studied using telemetric tools (Krop-Benesch et al. 2013).

In this study, in three pools of ten mosquitoes, at least five COI gene fragment sequence variants were present and five of those were sequences used as an example of identical or similar sequence variants (allele) distribution across Europe (Table 1 and Fig. 2). The sequences identical to three of the five sequence variants were found in material isolated from *Ae. vexans* and/or *C. capreolus* from Poland, Germany, Denmark, Hungary, France, and Spain (Table 1). Two remaining sequences amplified from *Ae. caspius* total DNA were unique, however, had 99% similarity to the DNA sequences previously detected in material isolated from *Ae. vexans* and/or *C. capreolus* in Denmark, Germany, and France. The COI gene fragment sequences [KM370867, KM370868] identified in an earlier xenomonitoring study conducted in Poland were identical to the sequence [KM452922] identified in this study and those sequences had 100% similarity to the sequences obtained from material isolated from *Ae. vexans* and/or *C. capreolus* in France, Denmark, Germany, and Hungary, which is indicated in Table 1. The high level of diversity of COI gene sequences in a single location, in a single country, and in one mosquito pool consisting of ten insects indicates that the parasite population in the investigated area had a high level of genetic diversity which in turn suggests that *S. tundra* was not introduced to the area recently. In a single W7 pool of ten mosquitoes collected in Wroclaw, there were at least two COI gene variants which were present also in Spain, France, Germany, Denmark, Central Poland, and Hungary (Fig. 2, Table 1). The presence of the same polymorphic sequences across southern, central, and northern Europe (Fig. 2) suggests that the parasite has been established in many European countries for a long time which is in agreement with previous findings (Angelone-Alasaad et al. 2016). The phylogenetic analyses of *S. tundra* COI gene sequences (Angelone-Alasaad et al. 2016) led to the conclusions that the parasite has spread from the south to the north of Europe and that *S. tundra* presence might have been undetected rather than absent in territories where the parasite was detected recently.

### Scarcity and diversity of molecular data

Once we found the link between the host *C. capreolus*, the vector *Ae. vexans*, and the *S. tundra* genotypes detected in both the host and the vector, the fact that two DNA sequences from Finland [DQ097309 and KP760209] which diverged from *S. tundra* COI gene sequences found in other countries (Angelone-Alasaad et al. 2016) were the only ones isolated from *R. tarandus* [DQ097309 and KP760209] drew our attention. The remaining sequences of *S. tundra* used for the phylogenetic analysis performed by Angelone-Alasaad et al. 2016 were isolated from either *Ae. vexans* [KF692103–KF692105] in the course of a xenomonitoring study conducted in Germany (Kronefeld et al. 2014), or from a worm specimen [AJ544874] whose geographical origin was not specified (Casiraghi et al. 2004). The highest similarity level between the *S. tundra* isolated from *R. tarandus* [DQ097309, KP760209] and the remaining sequences subjected to phylogenetic analysis in a study of European *S. tundra* isolates was only 98% (Angelone-Alasaad et al. 2016). These results might be an indication of the existence of genetic differences between *S. tundra* infecting *R. tarandus* and *C. capreolus*. We have found in GenBank a single COI gene sequence obtained from a worm isolated from *C. capreolus* in Finland [EF661849]; however, this sequence only partially overlapped with the sequences obtained from *C. capreolus* [KX599455, KX599456] isolated in Spain (Angelone-Alasaad et al. 2016) and one of the sequences from Finnish *S. tundra* isolates from *R. tarandus* [KP760209], which would explain why it had not been selected for phylogenetic analysis performed by Angelone-Alasaad et al. 2016. The scarcity of the sequences from Finnish *S. tundra* specimens from *C. capreolus* did not allow us to draw conclusions concerning the existence of distinct *S. tundra* genotypes infecting *R. tarandus* and *C. capreolus*; however, the fact that the *S. tundra* COI gene sequences found in mosquitoes and *C. capreolus* in Europe (excluding Finland) diverged from those found in *R. tarandus* indicated that there might be some bias for host preference depending on the *S. tundra* genotype. Molecular xenomonitoring of mosquitoes for filarial worms might become a useful tool for analysis of the definitive host and vector preference of filarial parasites if enough molecular data on the filarial genotypes present in the definitive hosts and mosquitoes from various geographical locations were available.

### PCR assay impact on xenomonitoring

In the course of our study, we also evaluated the applicability of the screening PCR designed for *Dirofilaria* sp. xenomonitoring (Table 2) to *S. tundra* xenomonitoring. All samples were subjected to the screening and the confirmatory PCRs in order to establish if the screening PCR was equally effective in the detection of both *S. tundra* and *Dirofilaria* spp. The applied screening PCR, despite the presence of mismatches between the primers RepIm-F and RepIm-R2 and the *S. tundra* COI gene (Table 2), detected all three samples positive for *S. tundra* in the confirmatory PCRs employing primers designed for *S. tundra* detection: RepIm-R0/Set-F0 and Set-F2/Set-R2 (Table 2). The screening PCR successfully used for *D. repens* xenomonitoring in central Poland (Masny et al. 2016) proved to be applicable to efficient *S. tundra* xenomonitoring and to have sensitivity of detection comparable to the two confirmatory PCRs designed for *S. tundra* detection (Table 2). Thus, the screening PCR with RepIm-F and RepIm-R2 primer pairs seems an universal tool for *D. repens*, *D. immitis*, and *S. tundra* detection. The mismatches with the *S. tundra* COI gene reduced the diagnostic sensitivity of the PCR with RepIm-F0 and RepIm-R0 (two out of three positive pools detected) and did not reduce the diagnostic sensitivity of the screening PCR (three out of three positive pools detected). What should be mentioned here is that the screening PCR has low specificity due to low stringency of PCR conditions; the specificity was traded for sensitivity—a common approach in screening assays whose results need to be confirmed by a specific assay. The problem of the impact of primer mismatches on the results of the PCR xenomonitoring experiments or lack of such influence is a complex issue extensively discussed previously (Masny et al. 2016) and only an experimental approach allows to determine the level of influence of mismatches on PCR sensitivity.

In this study, cross-reactivity of filariae COI gene detection by PCR assays was observed as in other studies (Czajka et al. 2012; Masny et al. 2016) and the cross-reactivity seems to be the rule rather than the exception in filarial COI gene detection. Only three of nine positive results of the screening PCR were confirmed in the confirmatory PCRs; the remaining six results might correspond to the detection of other filarial species than *D. repens*, *D. immitis*, or *S. tundra* or to the amplification of non-filarial DNA; as mentioned earlier in screening assays, the sensitivity can be increased at the cost of reduced specificity. We have found that the COI gene sequence of *S. tundra* isolated from *C. capreolus* from Denmark [KU508982] perfectly matched Set-Fs/Set-R2 primer pair and has a single mismatch with RepIm-R0 primer from Set-F0/RepIm-R0 primer pair. Thus, it cannot be excluded that the PCR assay with Set-F0/RepIm-R0 primer pair would have reduced sensitivity or would fail to detect some *S. tundra* genotypes, and the limited number of sequences of *S. tundra* COI genes present in GenBank might not reflect the real diversity of these sequences which reduces the value of primer specificity predictions based on GenBank similarity searches. Therefore, we would suggest that those interested in very accurate estimations of mosquito infection rates with *S. tundra* or other filarial nematodes, by PCR xenomonitoring, to use at least two species-specific PCR assays in mosquito screening and to perform sequencing of the PCR products for unequivocal species determination. We would like to emphasize that the assays allowing efficient amplification of filarial DNA from vertebrate tissues or worm tissue can be of low applicability to mosquitoes, which was shown previously (Masny et al. 2016). Therefore, it would be necessary to establish which assays are applicable to both worm and mosquito extracted DNA in order to collect same-length sequences to allow phylogenetic analyses such as those performed by Angelone-Alasaad et al. 2016. Summarizing, the simultaneous analysis of the phenology, feeding preferences, molecular diagnostics results, and xenomonitoring data may contribute to better understanding of *S. tundra* biology, its vector biology, and setariosis epidemiology. Further studies in Europe would be desirable for better recognition of the vector capacity of the different mosquito species as well as the *Setaria* pathogen ecology. Establishing standardized sets of molecular assays for xenomonitoring and worm analysis would be beneficial for interstudy comparisons and phylogenetic analyzes based on DNA sequences.
